# Supplementary material for: Microbe-mediated host defence drives the evolution of reduced pathogen virulence
Source: Nat Commun. 2016 Nov 15;7:13430. doi: 10.1038/ncomms13430 (PMC5116080; doi:10.1038/ncomms13430)
Supplement: Supplementary Information — Supplementary Figures 1-2 and Supplementary Tables 1-2 [file ncomms13430-s1.pdf]

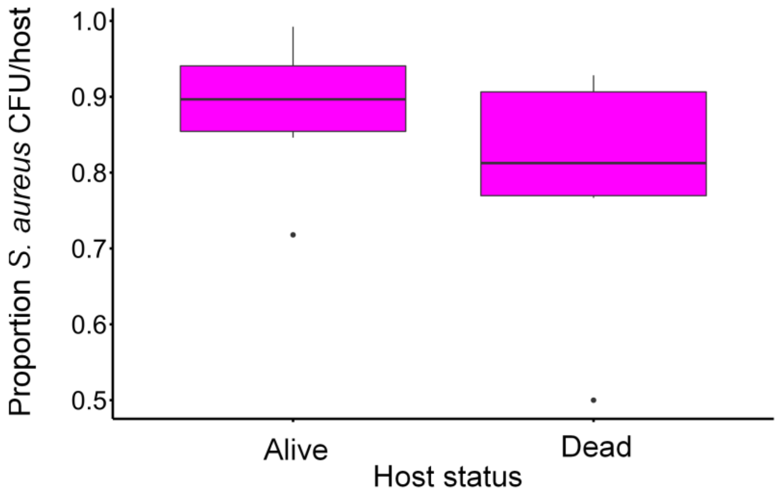

Supplementary Figure 1. The proportion *S. aureus* CFU of the total CFU (*S. aureus* + *E. faecalis* CFU) per host in worms alive or dead at 24 hours of exposure. Two sample t-test: t = 1.22, df = 10, P= 0.25. Sample size for each treatment: 6 biological replicates.

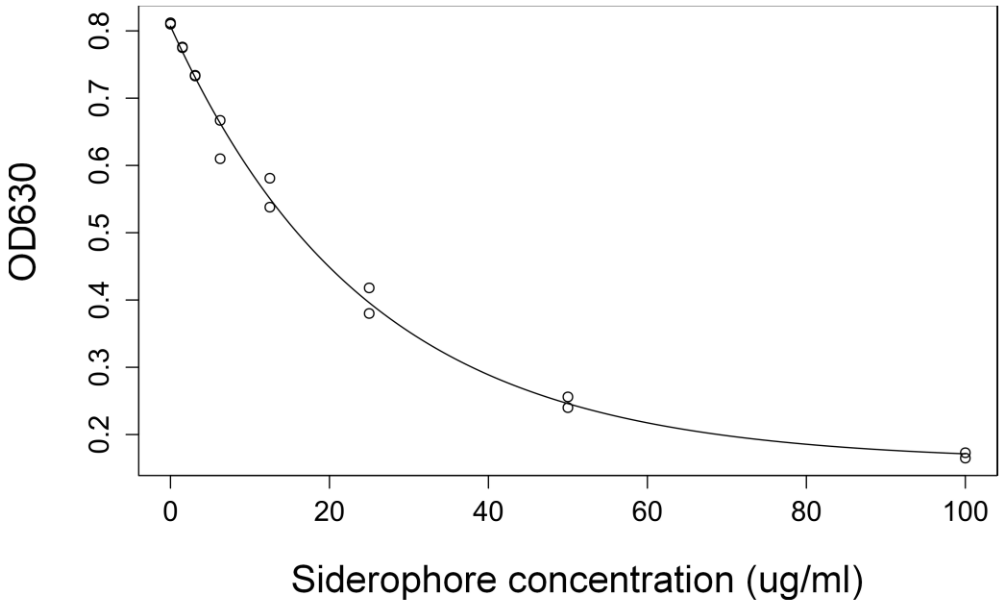

Supplementary Figure 2. The relationship between OD630 and siderophore concentration (ug ml^{-1}) *in vitro.* Nonlinear (weighted) least-squares estimates of the parameters of a nonlinear model: OD = 0.1599 - -0.6471*exp(-0.04*concentration). Sample size: 2 biological replicates for each concentration.

Supplementary Table 1. Statistical results listed by figure

Figure	Statistical results
2a	**Quasibinomial GLM:** F=41.96, df=2, P= 5.193e-09<br>Tukey contrasts:<br>*S. aureus* vs. *E. faecalis*: P<1e-04<br>*S. aureus* vs. co-colonisation: P<1e-04<br>*E. faecalis* vs. co-colonisation: P=0.025<br>Sample size for each treatment: 10 biological replicates.<br>Experiment replicated twice.
2b	**Welch two sample t test:** t=-4.5, df=16.2, P=0.0003<br>95 percent confidence interval:<br>-81755.14 -29842.08<br>Sample size for *S aureus*: 16 biological replicates.<br>Sample size for co-infection: 18 biological replicates.<br>Experiment replicated 3 times.
2c	**Two sample t test:** t=-2.8, df=24, P=0.009.<br>95 percent confidence interval:<br>-14421.823 -2292.925<br>Sample size for *E faecalis*: 11 biological replicates.<br>Sample size for co-infection: 15 biological replicates.<br>Experiment replicated twice.
4a	**Quasibinomial GLM:** F=25.7, df=2, P=4.627e-05<br>Tukey contrasts:<br>Coevolution vs. Ancestor: P<0.001<br>Coevolution vs. Single evolution: P<0.001<br>Ancestor vs. Single evolution: P=0.035<br>Sample size for each treatment: 5 biological replicates (average of 2 technical replicates).<br>Experiment replicated twice.
4b	**Pearson's product-moment correlation:** t = 3.38, df = 9, p= 0.008. R^2=0.56<br>95 percent confidence interval:<br>0.2682905 0.9303416<br>Sample size for virulence data: as fig.4a but all ancestral replicates averaged to 1 biological replicate.<br>Sample size for growth rate data: 5 biological replicates (average of 3 technical replicates) for evolved bacteria. 1 biological replicate for ancestral bacteria (average of 8 technical replicates). Anomalous technical replicate points identified and removed using the Dixon test.<br>Experiment replicated twice.
4c	**ANOVA:** F=6.55, df=2, P=0.012<br>Tukey contrasts & 95% family-wise confidence level:<br>diff lwr upr p value<br>Ancestor vs Coevolution: -16106.2 -31087.38 -1125.017 0.035<br>Single evolution vs Coevolution: -18791.0 -33772.18 -3809.817 0.015<br>Single evolution vs. Ancestor: -2684.8 -17665.98 12296.383 0.883<br>Sample size for each treatment: 5 biological replicates (1 technical replicate).<br>Experiment replicated once.

<table>
<tr><td>5a</td><td>ANOVA: F=6.681, df=2, P=0.01
<u>Tukey contrasts & 95% family-wise confidence level:</u>
diff lwr upr p value
Coevolution vs. Ancestor: -0.531180 -0.94121802 -0.121142 0.01
Single Evolution vs. Ancestor: -0.107136 -0.51717402 0.302902 0.8
Single Evolution vs Coevolution: 0.424044 0.01400598 0.834082 0.04
<u>Sample size</u> for each treatment: 5 biological replicates (average of 2 technical replicates).
Experiment replicated twice.</td></tr>
<tr><td>5b</td><td>Pearson's product-moment correlation: t = 2.8, df = 9, P=0.02. R^2=0.5.
<u>95 percent confidence interval:</u>
0.1441149 0.9106005
<u>Sample size</u> for virulence data: as fig.4a but all ancestral replicates averaged to 1 biological replicate.
<u>Sample size</u> for siderophore data: as fig.5a but all ancestral replicates averaged to 1 biological replicate.</td></tr>
<tr><td>7a</td><td>Pearson's product-moment correlation: t=6.1, df=2, P=0.026. R^2=0.95
<u>95 percent confidence interval:</u>
0.2052983 0.9994809
<u>Sample size</u>: 1 biological replicate for each concentration (average of 2 technical replicates).
Experiment replicated once.</td></tr>
<tr><td>7b</td><td>ANOVA: F=9.2, df=3, P=0.0009
<u>Dunnett contrasts:</u>
Coevolution S. aureus & ancestral E. faecalis vs. ancestral E. faecalis: P=0.77
Single evolution S. aureus & ancestral E. faecalis vs. ancestral E. faecalis: P=0.023
Ancestor S. aureus & ancestral E. faecalis vs. ancestral E. faecalis: P<0.001
<u>Sample size</u> for each treatment: 5 biological replicates (1 technical replicate).
Experiment replicated once.</td></tr>
<tr><td>7c</td><td>Control ANOVA: F=8.1, df=2, fdr corrected P=0.019
<u>Tukey contrasts & 95% family-wise confidence level:</u>
diff lwr upr p value
Ancestor vs Coevolution: 212500000 4318164 420681836 0.046
Single evolution vs Coevolution: 290000000 81818164 498181836 0.009
Single evolution vs. Ancestor: 77500000 -130681836 285681836 0.57
+Fe^{3+} ANOVA Treatment: F=3.05, df=2, fdr corrected P=0.092.
<u>Sample size</u> for each treatment of both ANOVAs: 5 biological replicates (1 technical replicate).
Anomalous points identified and removed using the Dixon test.
Experiment replicated once.</td></tr>
<tr><td>Supp 1</td><td>Two sample t-test: t = 1.22, df = 10, P= 0.25.
<u>95 percent confidence interval:</u>
-0.07642428 0.26070806
<u>Sample size</u> for each treatment: 6 biological replicates. Experiment replicated once.</td></tr>
<tr><td>Supp 2</td><td>Nonlinear (weighted) least-squares estimates of the parameters of a nonlinear model:
OD = 0.1599 - -0.6471*exp(-0.04*concentration)
<u>Parameters:</u>
Estimate Std. Error t value Pr(>|t|)
a 0.159890 0.014898 10.73 7.88e-08 ***
b -0.647120 0.015333 -42.20 2.68e-15 ***
c 0.040329 0.002729 14.78 1.66e-09 ***
Signif. codes: 0 ‘***’ 0.001 ‘**’ 0.01 ‘*’ 0.05 ‘.’ 0.1 ‘ ’ 1
Residual standard error: 0.01949 on 13 degrees of freedom</td></tr>
</table>

	Number of iterations to convergence: 4<br>Achieved convergence tolerance: 2.732e-07<br>Sample size: 2 biological replicates for each concentration.<br>Experiment replicated once.

Supplementary Table 2. Mutations found in *S. aureus* listed by treatment and replicate population. Locus tag is given when mutation occurs in a coding region. Otherwise, the coordinate is given. Mutation abundance is the percentage of 40 sampled clones that contain the mutation in the replicate population. Only mutations present in >20% clones are listed to control false positives at the expense of low frequency true positives. Gene names and putative gene function are given where known, along with a reference. A proposed link with siderophore activity is listed where identified. Mutation type: S=synonymous. N=non-synonymous. *=stop gain. */= stop loss.

Treatment	Replicate	Locus tag/ coordinate	Mutation type	Mutation abundance (%)	Gene name/annotation	Putative function	**Putative** link with siderophores	Reference (PMID)
Coevolution	1	SAS_RS09425	N	95	Protein export protein PrsA Foldase.	A facultative facilitator of protein secretion or extracellular folding. Cell wall biosynthesis.	Excretion or folding of proteins involved in iron acquisition	22526301 21765893
	2	SAS_RS00600	*	98	Hypothetical protein.	ABC transporter superfamily.	Transport of substrates involved in iron acquisition	25414356
		549806		100	Between pyrimidine nucleoside transporter NupC & transcriptional regulator CtsR.			
		SAS_RS02825	N	30	Glycosyl transferase family 1.	Cell wall biosynthesis.		17237164
		SAS_RS02905	S	100	Uracil-DNA glycosylase.	DNA base-excision repair.		2836397
		SAS_RS04210	N	23	Cysteine desulfurase.	iron-sulphur cluster biosynthesis.		11577100
		SAS_RS13205	N	98	Holin-like protein CidB.	Cytolysis. Cellular suicide in response to cellular stress or damage.		12670989 14617136
	3	629811		95	Between octanoyl-[GcvH]:protein N-octanoyltransferase and mevalonate kinase.			
		SAS_RS05930	N	43	Penicillin-binding protein.	Cell wall biosynthesis.		3882429
		1267927		30	Between DNA topoisomerase 1 and TrmFO.	TrmFO: oxidoreductase flavoprotein.	TRmFO: Siderophore-iron reductase	

Treatment	Replicate	Locus tag/ coordinate	Mutation type	Mutation abundance (%)	Gene name/annotation	Putative function	**Putative** link with siderophores	Reference (PMID)
		SAS_RS07345	N	98	Sulphite reductase subunit alpha.	Sulphite to sulphide reduction. Iron ion binding. Oxidoreductase. ferrisiderophore reduction. Hydrogen sulphide biosynthesis. Sulphate assimilation.	Siderophore-iron reductase	7657631
		1839866		28	Between MarR family transcriptional regulator and lysophospholipase.			
		SAS_RS11185	N	43	Methicillin resistance protein FmtB.	Cell wall biosynthesis.		17951380 10896508
		SAS_RS13665	N	55	Hypothetical protein.	No conserved domains identified.		
	4	SAS_RS13620	*/	80	Anaerobic ribonucleoside-triphosphate reductase activating protein.	Oxidoreductase. Iron-sulfur cluster binding.	Siderophore-iron reductase	10821845
	5	SAS_RS03320	N	93	Membrane protein.	Solute carrier families 5 and 6-like. Transports inorganic ions, sugars, amino acids.	Transport of substrates involved in iron acquisition	25414356
		SAS_RS12635	N	25	Glycerate kinase.	Glycine metabolism.	Ferrichrome siderophore is made of glycine residues	

Treatment	Replicate	Locus tag/ coordinate	Mutation type	Mutation abundance (%)	Gene name/annotation	Putative function	**Putative** link with siderophores	Reference (PMID)
Single Evolution	1	SAS_RS06355	N	28	RIP metalloprotease RseP.	Coordinating gene transcription during extracytoplasmic stress response.		12073657
		SAS_RS09685	N	50	2-hydroxyacid dehydrogenase.	Oxidoreductase.		17049749
	2	10884		33	Between carbohydrate kinase and histidine ammonia-lyase.			
		SAS_RS01275	N	35	Ribose transporter RbsU.	Sugar transport across bacterial membrane. Uptake of ribose.		10941799
		SAS_RS03290	N	28	Ferrichrome ABC transporter permease.	Part of the ABC transporter involved in iron-siderophore import.	Transport of substrates involved in iron acquisition	3020380
		SAS_RS03845	*	30	Helicase.	ATP-dependent RNA or DNA unwinding.		
		SAS_RS04065	S	25	Thermonuclease.	Catalyses the hydrolysis of both DNA and RNA.		6307819
	3	626196		25	Between hypothetical protein and heme-binding protein.		Heme-binding protein; iron acquisition	
		SAS_RS05650	N	28	Heme uptake system protein IsdE.	ABC transport of heme, siderophores and metal ions.	Transport of substrates involved in iron acquisition	15240116
		1985301		33	Between aspartyl/ glutamyl-tRNA(Asn/Gln) amidotransferase subunit C and sodium:proline symporter.			
		SAS_RS11115	N	38	Membrane protein.	No conserved domains identified.		

Treatment	Replicate	Locus tag/ coordinate	Mutation type	Mutation abundance (%)	Gene name/annotation	Putative function	**Putative** link with siderophores	Reference (PMID)
		SAS_RS12235	N	33	DNA-binding response regulator.	Contains a singal receiver domain, an effector domain and a DNA-binding response regulator.		
		SAS_RS13800	N	30	Surface anchored protein.	No conserved domains identified.		
	4	No mutations >20%						
	5	SAS_RS12000	N	23	RpiR family transcriptional regulator.	Transcription factor.		
